# Corneal Biomechanical Properties in Different Ocular Conditions and New Measurement Techniques

**DOI:** 10.1155/2014/724546

**Published:** 2014-03-04

**Authors:** Nery Garcia-Porta, Paulo Fernandes, Antonio Queiros, Jose Salgado-Borges, Manuel Parafita-Mato, Jose Manuel González-Méijome

**Affiliations:** ^1^Clinical & Experimental Optometry Research Lab, Center of Physics (Optometry), School of Sciences, University of Minho, Gualtar, 4710-057 Braga, Portugal; ^2^Grupo de Investigación en Superficie Ocular y Lentes de Contacto, Departamento de Cirugía (Oftalmología), Universidad de Santiago de Compostela, 15782 A Coruña, Spain; ^3^Department of Ophthalmology, Centro Hospital de Entre Douro e Vouga, Santa Maria da Feira, Portugal; ^4^Department of Ophthalmology, Hospital Escola, Universidade Fernando Pessoa, Gondomar, Portugal

## Abstract

Several refractive and therapeutic treatments as well as several ocular or systemic diseases might induce changes in the mechanical resistance of the cornea. Furthermore, intraocular pressure measurement, one of the most used clinical tools, is also highly dependent on this characteristic. Corneal biomechanical properties can be measured now in the clinical setting with different instruments. In the present work, we review the potential role of the biomechanical properties of the cornea in different fields of ophthalmology and visual science in light of the definitions of the fundamental properties of matter and the results obtained from the different instruments available. The body of literature published so far provides an insight into how the corneal mechanical properties change in different sight-threatening ocular conditions and after different surgical procedures. The future in this field is very promising with several new technologies being applied to the analysis of the corneal biomechanical properties.

## 1. Introduction

Corneal biomechanics is a branch of science that studies deformation and equilibrium of corneal tissue under the application of any force [1]. The structure and hence the properties of a soft tissue, such as the cornea, are dependent on the biochemical and physical nature of the components present and their relative amounts. The mechanical properties of a tissue depend on how the fibres, cells, and ground substance are organized into a structure [2]. Collagen and elastin are responsible for the strength and elasticity of a tissue, while the ground substance is responsible for the viscoelastic properties. All these terms are important because the cornea is considered a viscoelastic material and some devices try to measure and even differentiate between the different components of the biomechanical behavior of the living corneal tissue [3]. In the specific case of the human cornea, collagen in Bowman's layer and stroma accounting for over 80% of the dry weight of the cornea would be the major contributor to corneal elasticity. The ground substance, formed mostly by proteoglycans and keratocytes or fibroblasts, would provide the viscous behaviour. The corneal epithelium accounting for 10% of the central corneal thickness could also contribute to the viscous behaviour. It is important to bear in mind that the corneal epithelium is easily deformable and is the reference surface for most of the biomechanical corneal measurements.

Over the past two decades, researchers have developed a variety of techniques that can alter corneal surface for refractive purposes or even for halting disease progression in corneas with mechanical decompensation. Beside geometric corneal parameters, the additional influence of the biomechanical corneal properties has received little attention, mostly because of the lack of appropriate *in vivo* measurement techniques. However, in recent years, increasing interest has arisen in corneal biomechanics to predict corneal response to surgical or therapeutic interventions and to assist in the detection of early keratoconus [4–6]. Additionally, increasing interest has also arisen in corneal biomechanical properties and glaucoma once corneal biomechanics have been shown to influence intraocular pressure (IOP) measurements and may be also indicative of ocular globe biomechanics that could also be predictive of glaucoma susceptibility [7].

Corneal biomechanics have been assessed in *in vitro* studies by measuring stress-strain and Young's modulus in isolated corneas [8]. In the recent years, two devices have been marketed: the Ocular Response Analyser (ORA, Reichert, Depew, NJ) since 2005 and the Corneal Visualization Scheimpflug Technology (Corvis ST, Oculus, Wetzlar, Germany) since 2011. Many studies covering a wide range of topics have been conducted and published using the ORA.

The aim of the present review is to provide an overview of published results on corneal biomechanics obtained with ORA under different ocular and systemic conditions. Knowledge accumulated to date on this field will potentially help the ophthalmic community to gain a better understanding of the changes that the corneal tissue undergoes during different ocular and systemic conditions as well as to predict the outcomes of therapeutic and refractive therapies. New technologies under development will also be discussed briefly since there is currently a wide range of instrumentation under development to provide a better understanding of the biomechanical nature of the cornea and its implications in visual care, with particular relevance to the detection and management of sight-threatening conditions.

## 2. Biomechanical Descriptors and Their Physical Meaning

To better understand the results of corneal biomechanical measurements, it is important to remember the meaning of some corneal properties such as elastic, viscous, or viscoelastic response, hysteresis, and stiffness, among other concepts.

**Figure 1:**
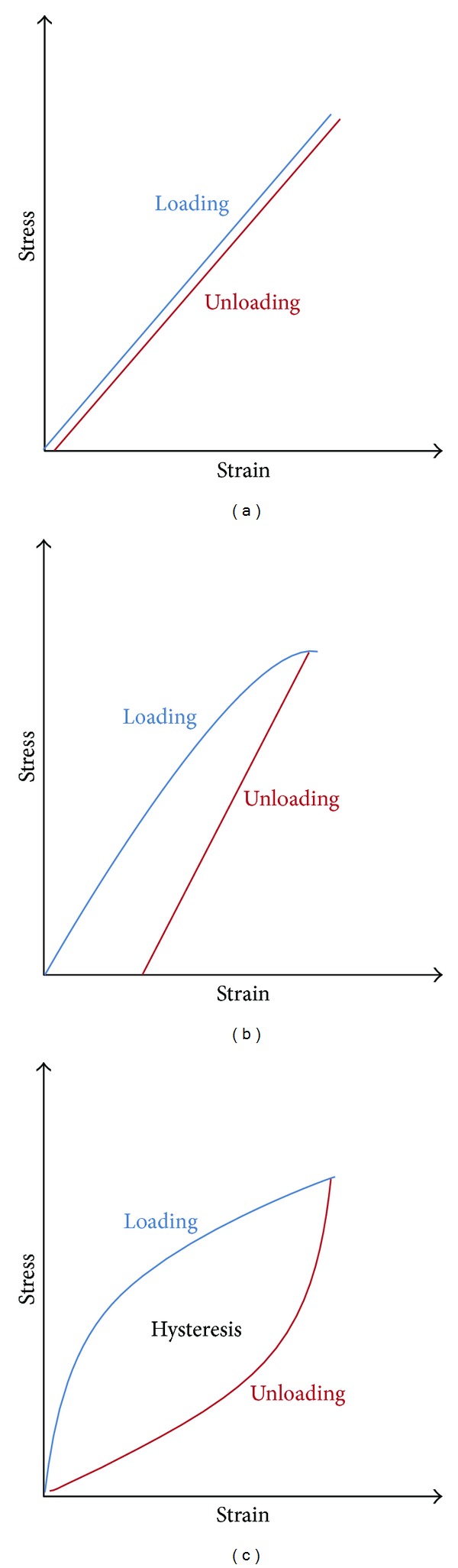
Stress-strain response diagrams of different materials showing elastic (a), plastic (b), and viscoelastic behaviour (c).

- The *elastic response* of a material is attributed to the instantaneous and reversible deformation under an external load [2]. In elastic materials, the deformation is proportional to the force applied and it is recovered instantly upon unloading. Thus, the stress-strain relationship would be a straight line [9].  Figure 1(a) shows the typical stress-strain diagram of an elastic material. The constant of proportionality between stress and strain is the elastic modulus, also called *Young's modulus*. Young's modulus is defined as the ratio of the *stress* (load per unit area) and the *strain* (deformation/displacement per unit length) [10]. A high modulus indicates a *stiffer material* (i.e., not easy to bend). This also leads us to the definition of *resistance*, which is the capacity of a material to hold stress without deformation.
- Corneal Young's modulus, measured *in vitro*, varies from 0.1 to 57 MPa [8, 11–20] that might be explained by variations in testing conditions and methods used. More recently, Hamilton and Pye [21], using the Orssengo-Pye algorithm, reported on 100 healthy eyes with mean Young's modulus being 0.29 ± 0.06 MPa (range 0.13 to 0.43 MPa). Modulus was positively correlated with the IOP measured with GAT, assuming that Young's modulus itself affects the IOP measurement.
- A material shows a *viscous behaviour* when the deformation velocity is faster than the relaxation rate. The slow relaxation is due to configurational rearrangement of the material during deformation [2].
- *Viscoelastic materials* exhibit elastic and viscous behaviour at the same time, so they present characteristics of elastic and viscous materials [2].  Figure 1(c) shows the typical stress-strain diagram of a viscoelastic material. Their particular characteristics make it possible to define characteristic properties including one known as “hysteresis”.
  - *Hysteresis* in viscoelastic materials under periodic loading and unloading, curves in the stress-strain diagram (Figure 1(c)) are not coincident with each other; the gap between them is called hysteresis [22].
  - The *energy stored* over one full loading and unloading cycle in a material is zero since the material returns to its initial configuration (elastic behavior). The area within the hysteresis loop represents the *energy per volume dissipated* in the material per cycle [23].

### 2.1. Parameters Derived from Ocular Response Analyzer

**Figure 2:**
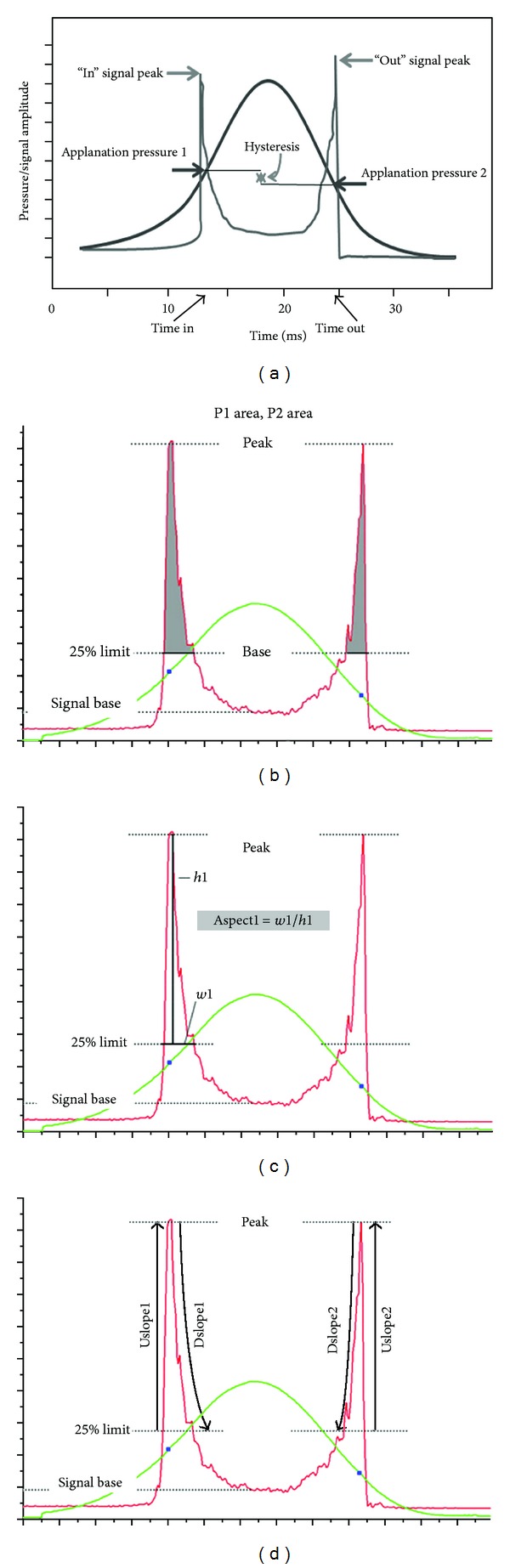
Applanation and pressure plot as determied by the ORA (Ocular Response Analyzer). See text for definition of the different variables indicated in the graphs.

The ORA is a noncontact tonometer introduced in clinical practice in 2005 [3]. It uses a rapid air pulse to indent the cornea and an electrooptical system to record corneal deformation. It records mainly two applanation measurements: one while the cornea moves inward, reaching a first applanation, when the first pressure (*P*_1_) is registered and the other as the cornea recovers from a slight concavity as the air pump decreases pressure at an inverse rate so that the cornea moves outward passing through a second applanation (*P*_2_). Therefore, these two values, *P*_1_ and *P*_2_, indicate the pressure necessary to flatten the cornea during the loading and unloading cycle (Figure 2).

Thus, below we define one by one the terms and parameters that are relevant to the understanding and interpretation of the outcomes obtained by the ORA according to the literature.

- *P*_1_ and *P*_2_: air pressures corresponding with the two applanation states of the cornea.
- *Corneal hysteresis* (CH) is considered an *indicator of corneal viscosity* and is obtained by the difference between the 2 pressures: CH = *P*_1_ − *P*_2_ [3].
- The *corneal resistance factor* (CRF) is considered an *indicator of the overall resistance of the cornea* and is expressed by the equation: CRF = (*P*_1_ − 0.7∗*P*_2_) [24]. It is significantly correlated with central corneal thickness (CCT) and Goldmann applanation tonometry (GAT) [3]. It has been also suggested that the CRF could be mainly related to *the elastic properties* of the cornea [25]. Other authors suggested modifications on the original formula to CRF = *k*_1_∗(*P*_1_ −0.7∗*P*_2_) + *k*_2_, where *k*_1_ and *k*_2_ are constants [26, 27]. Moreover, some authors evaluated the difference between CH and CRF, but the meaning of this “new” parameter [28, 29] is not clear.
- IOPg is an IOP value equivalent to GAT, which is an average of the two pressure values measured by ORA, *P*_1_, and *P*_2_ and obtained by the following equation: IOPg = (*P*_1_ + *P*_2_)/2 [24].
- IOPcc is a new IOP value called *Corneal Compensated IOP* and is obtained by the equation IOPcc = *P*_1_ − 0.43*P*_2_. It is less affected by corneal properties than by the IOP obtained with other tonometers and it is not correlated with the CCT [24] but it is correlated with CH [30, 31].
- *Corneal constant factor* (CCF) is claimed to be an IOP-independent corneal factor introduced by Kotecha et al. [26] and was derived from the changes of *P*_1_ and CH for every 1 mm Hg of change in GAT IOP. It describes an IOP-independent biomechanical property that increases with thicker CCT and decreases with aging and yet explains more of the interindividual variation in GAT IOP than does CCT. It is very similar to CRF proposed by Reichert and is expressed by the equation: CCF = *P*_1_ −0.79∗*P*_2_.

The deformation signal waveform produced by the corneal deformation signal (characteristic shape illustrated in Figure 2) can provide a unique description of each eye. Further analysis of the waveform signal delivered by the electrooptical system of the instrument has provided more parameters with potential interest to allow a refined evaluation of the corneal properties [32]. Recently, 37 new parameters were derived from the new ORA software allowing a detailed analysis of the deformation signal waveform. Each one of these parameters describes a morphological feature of the waveform and 23 parameters are derived from the upper 75% of applanation peak height and 14 are derived from the upper 50% of the applanation peak height (Figure 2). These new parameters are defined in Appendix A. Most of these parameters depend on *P*_1_ and *P*_2_ defined at the beginning so, in some way, these parameters could be intrinsically linked and their clinical significance and the manner in which these individual parameters represent biomechanical properties are currently unknown. Several studies have investigated the clinical relevance of the new waveform parameters and reported that they could be more useful in diagnosis and prognosis after refractive surgery, and as stated in the following sections, some of these parameters seem to be promising as being more sensitive than others to detect corneal changes in specific corneal  conditions [28, 33–36].

## 3. Factors Affecting Corneal Biomechanical Properties

The possibility to evaluate the biomechanical properties of the cornea provides a new diagnostic tool that will allow detecting differences in corneal biomechanics between normal eyes and pathological eyes and eventually detecting weaker corneas at a subclinical state before they evolve in some kind of ectasia or avoiding postsurgical ecstatic disease. Since the introduction of ORA in clinical practice, many research studies have been conducted looking for associations between both CH and CRF and different parameters like age, corneal thickness, IOP, progress of glaucoma, or presence and severity of a given condition such as keratoconus [37]. According to Luce [3], corneas with low CH are less capable of absorbing energy than normal eyes and they may be candidates for several ocular diseases. Moreover, low CRF indicates that the overall corneal rigidity is lower than normal.

Table 1 shows results of different studies on healthy eyes. It is observed that both CH and CRF vary in a rather wide range in the normal population and that a comparison between studies for both parameters is difficult.

### 3.1. AGE

Several studies investigated the associations between changes in corneal biomechanical parameters and aging. Several studies found no significant differences in ORA measurements with ageing [38–42]. Lim et al. [40], in a study with 271 children, reported that CH and CRF did not vary significantly with age but the range of ages was quite narrow. Notwithstanding, as the authors observed, the values of CH and CRF measured were slightly higher than those in other adult studies. The same was observed by Kirwan et al. [38] in children and adolescents who also found no correlation between age and CH. However, when compared with other studies, the values of CH were again slightly higher. On the other hand, some studies have shown that CH significantly decreases with age [4, 26, 43–45]. Kamiya et al. [43] evaluated 204 eyes of healthy subjects and found a small but statistically significant negative correlation between CH and CRF with age without significant differences in central corneal thickness (CCT) or IOP across the sample. Ortiz et al. [4] only found significant differences in CH and CRF between subjects younger than 14 and older than 60, but a linear correlation between these two biomechanical parameters and ageing did not exist. Kotecha et al. [26] observed a reduction in CH of approximately −0.28 mm Hg/decade, while Foster et al. [46] found that the CRF declined significantly with age at a rate of −0.31 mm Hg/decade, as did CH by −0.34 mm Hg/decade.

In any case, due to the potential limitations of these studies, we should be careful to extrapolate their results to the general population. For instance, in one of these studies the sample was quite limited, with only fifteen subjects [44]. In another study, the changes are possibly confounding because of the proportion of the participants affected by ocular hypertension, glaucoma, or pigment dispersion syndrome [26]. Due to age-related changes in corneal structure such as an increase in collagen fibril diameter or intermolecular Bragg spacing [47], it would be expected that corneal biomechanical properties change with ageing. In fact, *ex vivo* studies have shown an increase in corneal stiffness with ageing [48] and that Young's modulus of the human cornea approximately doubles between the ages of 25 and 100 [49]. Considering this, if the CRF is a real indicator of corneal rigidity, it should change with ageing as well. Nevertheless, due to the intersubject variability and the differences among the results published in the different studies, we cannot conclude, based on present data, that CH and CRF parameters are able to confirm *in vivo* and in the clinical routine the expected changes towards a stiffening of the cornea.

### 3.2. Central Corneal Thickness (CCT)

Several studies investigated the potential effect of CCT on the biomechanical properties of the cornea measured with ORA. In fact, many studies reported a positive correlation between CCT and CH [3, 24, 30, 42, 50, 51] and also with CRF [24, 30, 40, 44, 51]. These studies included healthy subjects from different races/ethnicities and with a wide range of age. Recently, Leite et al. [52] found that black subjects had lower CH values compared to white subjects, but although they attributed those differences in CH to differences in corneal thickness between the two groups, they did observe a statistical trend towards lower CH among black subjects even when adjusting for CCT. A similar result was observed in a study with a strong statistical power by Haseltine et al. [53].

These results are in agreement with the expected response because a thinner cornea will be easier to deform, while a thicker healthy cornea containing more collagen fibers and ground substance will present a higher resistance against deformation and a higher damping capacity. Consequently, the stronger the corneal tension, the faster the cornea recovers its original position following deformation. CCT also suffers circadian changes and this might affect the biomechanical properties measured. There are a couple of articles where the 24-hour changes of CCT and corneal biomechanical properties were analysed [44, 54]. Despite a significant change between the nocturnal and diurnal CCT values, a significant change in the CH and CRF was not observed. These results could be explained considering that nocturnal CCT increase is related to increase in corneal hydration instead of collagen fibril or ground substance changes that would potentially reflect more directly on the biomechanical behaviour of the cornea.

### 3.3. Refractive Error and Axial Length

The degree of myopia is correlated with axial length (AL). Furthermore, it has been claimed that longer eyes are associated with flat corneal curvature and thinner corneas [55]. Furthermore, longer eyes had thinner sclera walls and possible thinner choroidal structure. In this way, according to previous section, if the highly myopic eyes have thinner corneas and if corneal biomechanical response might be somewhat related to the whole-eye biomechanical response, it would be expected that that more myopic eyes have lower CH values. It has been the goal of some studies to test the hypothesis that the weaker scleral structure of highly myopic eyes might be reflected and quantified in some way through the biomechanical analysis of the cornea.

Studies performed in Chinese subjects [41, 56] and Caucasian subjects [57] with a wide range of refractive errors observed a significant negative correlation between CH and myopia. Shen et al. [41] found lower CH in highly myopic eyes (−9 D) and no statistically significant differences in CH between emmetropes and low myopes (+0.25 to −2.75 D) or moderate myopes (>−3.00 to −6.00 D). Similar results were reported by Jiang et al. [56], but the reason of this decrease was not fully explained. However, although variation was not observed neither in CCT nor in CRF among subjects with different myopia degree, it is possible that the changes are related to the different characteristics of the cornea rather than weaker sclera structure which is characteristic of the highly myopic eyes. Recently, Xu et al. [58], in a study of subjects with myopic anisometropia, reported a significant lower CH in high myopic eyes compared to contralateral normal eyes. In this study, the difference in AL between the two eyes that resulted in anisometropia and CH was correlated with AL and CCT in high myopic eyes, whereas in the contralateral eyes, it was only correlated with CCT. Additionally, since differences in IOPg and IOPcc between the high myopic and contralateral eye were not observed, the authors suggest that the difference in AL does not occur by virtue of higher IOP, but it is possible that eyes with lower CH and thinner scleral structure may be easier to elongate [58, 59]. However, these studies do not permit elucidation if the lower CH and thinner scleral structure are the cause or the consequence of the increasing myopia of those eyes.

Yet, despite above studies indicate that the mechanical strength of the anterior segment of the eye is somehow compromised in high myopia, other previous studies did not show a correlation between refractive error and ORA measurements [40, 59, 60]. The study conducted by Radhakrishnan et al. [60] evaluated 95 normal myopic adult subjects (19 to 48 years) and found that CH was not significantly correlated with refractive error, while CRF showed a statistically significant but very weak correlation with spherical equivalent refractive error (*r* ^2^ = 0.04). However, the mean spherical refractive error was −1.78 ± 2.26 D and both parameters showed a considerable scatter across the sample under analysis.

### 3.4. Intraocular Pressure (IOP)

The Goldmann applanation tonometer (GAT) is the reference method to measure the IOP but when the IOP is measured with GAT it is assumed that the cornea is uniformly thick and perfectly elastic and behaves like a thin and perfectly flexible membrane [61]. Actually, none of these assumptions applies to the anatomical structure and physical behaviour of the living cornea under applanation forces. The pressure required to applanate the cornea depend on the IOP and the corneal rigidity [42], and it is well known that the IOP measures are influenced by CCT with thicker corneas requiring stronger force to applanate than thinner corneas, independent of IOP [10]. Many published articles have proposed linear correction factors to convert measured IOP into “true” IOP, on the basis of CCT. However, reported correction factors are different and mostly dependent on the population under study and can lead to corrections that may be wrong in magnitude and in direction such as correcting down when the true pressure is actually higher [62]. In fact, corneal biomechanical properties seem to be stronger predictors of IOP measurement error than does CCT alone [10]; this might explain the success of the ORA over the last 8 years for the IOP measurement in several corneal conditions.

IOPg provided by ORA is analogous to standard noncontact tonometry IOP measurements whereas IOPcc takes into account the biomechanical properties and is independent of the CCT as explained above. Although some studies find no mean difference between GAT and both ORA IOP measurements [24, 31, 63], other studies found poor agreement between GAT and IOPg and IOPcc with a significant overestimation of IOPg and IOPcc compared to GAT [27, 64]. Medeiros and Weinreb [31] found that GAT IOP was significantly correlated with CCT and significantly influenced by CRF, while IOPcc was not, and similar results have been confirmed by others [27, 64, 65]. Therefore, the effect of CCT on IOP overestimation may be explained by CRF and the resistance against deformation of the cornea which is also higher in eyes with higher IOP values [27]. In contrast, some studies reported the lack of association between CH and both GAT and IOPg [30, 64, 65], suggesting that CH is independent of IOP, while other studies suggest a relationship between CH and IOP. CH has been shown to decrease as the IOPcc increases [30, 46, 66, 67]. Kamiya et al. [66] found IOP as a significant explanatory variable relevant to CH, while González-Meijome et al. [68] found a significant correlation between changes in IOP and changes in CH during the day in healthy eyes. Also, CH has been shown to increase when IOP was lowered to normal range in patients with chronic primary angle-closure glaucoma [69].

Considering the previous results and despite some controversy, it is expected that in corneas with higher CH and higher CRF and therefore higher resistance to deformation, the values of GAT IOP or IOPg may be higher than the actual values and IOPcc could be a more reliable measure in those cases. The opposite might hold true in cases of lower CH and lower CRF where the actual IOP might be higher than actually measured by conventional methods. Again, IOPcc might provide a more realistic measure of the intraocular pressure.

### 3.5. Soft Contact Lens Wear

Reduced oxygenation of the cornea during contact lens (CL) wear is known to produce corneal edema that is reflected in an increase in corneal thickness (swelling). In fact, in a recent study, it was observed that the myopic subjects wearing soft contact lenses have higher values of CH and CRF than noncontact lens wearers [70]. The corneal swelling response with contact lens wear and eye closure averaged from *∼*3% to *∼*10% [71, 72] and some studies have analysed these effects on ORA measurements [70, 73, 74]. Lau and Pye induced corneal edema wearing soft contact lens for three hours and found no change in CH even with 13.1% corneal swelling, while CRF was elevated by a maximum of 0.6 mm Hg immediately after lens removal and was followed by a gradual recovery to normal values. Additionally, there were significant increases in IOPg but not in IOPcc and there were significant but weak correlations between changes of CCT and IOPg and IOPcc and CRF. Lau and Pye [74] found that CH and CRF respond to corneal swelling in dissimilar ways: CH was reduced by 0.6 mm Hg immediately after lens wear before returning to baseline, while CRF was elevated by a maximum of 0.6 mm Hg. In addition, the ability of CCT to predict both CH and CRF was significantly different between control and monocular closed-eye contact lens wear and the GAT overestimation observed is associated with an overall increase in CRF caused by small amounts of corneal swelling. Differences in the study population as well as in the amount of corneal swelling induced are likely contributors to the differences in the results between the two studies. However, the results suggest that ORA-generated parameters may be different in subjects with and without contact lens wear when significant amounts of edema are present. This kind of response, commonly observed in aphakic patients with overnight wear of thick CL, is not expected with regular use of silicone hydrogel contact lenses under daily wear conditions by patients within the normal range of refractive errors.

### 3.6. Orthokeratology

Orthokeratology (OK) is a technique that uses special gas permeable CL to temporarily reduce myopia by flattening the cornea. Therefore, the epithelial corneal thickness profile is changed and the cornea is significantly flattened by the use of these CL [75, 76] and the corneal biomechanical properties could be affected by these changes. Biomechanical properties of the cornea may help to understand the different responses to OK among different subjects. A study published in 2008 [5] investigated the changes of ORA measurements, CCT, and topography in subjects three hours after wearing OK lenses and three hours after removing the CL in order to assess the effect of corneal biomechanical properties on response (corneal flattening) and recovery (corneal steepening) during OK lens wear and after removal, respectively. The authors found that corneas with high values of CH showed a slower response and slower recovery to the OK treatment in the short-term treatment (3 hours of treatment). In another study, during short-term OK treatment, CRF was shown to decrease with increasing duration of lens wear, while there was no significant change in CH [77]. On the other hand, a significant decrease in CH and CRF was reported within the first week of OK treatment [78]. However, CRF and CH returned to original values and remained unchanged thereafter. According to the authors, the early reduction in CH and CRF may be due to a temporal response of reshaping of the corneal surface, rather than changes in the corneal microstructure. This may explain why there is a trend for CH and CRF to be reduced during the first month of treatment and after 1 year of treatment; when this is interrupted, CH and CRF show a trend to return to baseline values [79].

The knowledge of these associations could help to have a better predictability of the OK effect [5, 80] and then to choose the suitable patients to undergo OK treatment or to predict the speed of onset and recovery of the effect.

### 3.7. Refractive Surgical Treatments

#### 3.7.1. Refractive Surgery

Laser corneal ablation might have significant implications on corneal mechanical resistance. Several studies showed invariably a significant reduction of CH and CRF by about 1 to 3 mm Hg approximately after different laser refractive treatments [4, 6, 25, 30, 81–87]. The results from these studies are summarized in Table 2. Studies comparing different laser refractive techniques showed a higher decrease in both CH and CRF in LASIK eyes when compared with photorefractive keratectomy (PRK) [83]. Similar decrease in CH has been documented for LASIK and laser-assisted subepithelial keratectomy (LASEK) [6]. This biomechanical effect was correlated with deeper ablation because more central collagen and matrix material would be removed [4, 81] or with the potential effect of flap preparation that itself causes a reduction in both CH and CRF [82, 88, 89]. Ortiz et al. [4] found a moderate correlation between the refractive error correction and the change in CH (*r* = 0.5, *P* = .007) and CRF (*r* = 0.6, *P* = .001) in myopic LASIK, while a smaller decrease in CH and CRF was found in hyperopic LASIK eyes than in myopic LASIK and LASEK eyes, supporting the predominant effect of tissue ablation [89]. Gatinel et al. [88] found a reduction in both CH and CRF with microkeratome-assisted flap creation alone. Qazi et al. [82] found that despite similar changes in CH and CRF in the myopic LASIK and myopic LASEK groups, there were significantly greater postoperative changes in the ORA waveforms in the LASIK groups than in the LASEK group with the amplitude of Peak 1 being less reduced in the group of LASEK, suggesting that the creation of a flap has a greater effect on these waveform parameters than the depth or location of the stromal ablation. Similar results were reported by Franco and Lira [30] who found that, as a result of induced changes in viscous and elastic properties by LASIK, the time needed for the first applanation of the cornea (Time in) was higher in normal than in post-LASIK eyes and that the post-LASIK eyes needed more time to recover their shape (Time out parameter).

Studies reporting the time course of ORA parameters after different surgical techniques showed that the largest changes occurred within the first few weeks after surgery and then became nearly stable or even showed a slight recovery in the medium and longer term [84, 86, 87]. Surgically induced corneal ectasia is a rare complication of refractive surgery and is thought to be a result of biomechanical decompensation due to an insufficient residual stromal bed thickness after the surgery or when surgery is performed on unidentified subclinical keratoconic cornea. Thus, the possibility of using ORA parameters for assisting in the detection of corneas at risk has been very promising since the ORA was marketed. Although a low CH (<8 mm Hg) might be a predictive index of a preectatic conditions [3, 33], the overlap in the distribution of both CH and CRF values within the normal population does not support a role for CH and CRF measurement as single predictors to detect early ectasia or to predict its onset before surgery [91]. Instead, waveform analysis of ORA signals [33, 82, 92] has shown that the morphology of the signal may provide additional information. For instance, in a case of iatrogenic ectasia after LASIK, Kerautret et al. [33] found a lower Peak 1 height in the ectatic eye than in the fellow nonectatic eye, despite the similar CH and CRF values in the 2 eyes. These findings may suggest that a higher Peak 1 is associated with a stiffer cornea [82]. Considering that recent studies seem to indicate that the new ORA parameters represent a significant improvement over CH and CRF alone, more research is needed to confirm and improve the sensitivity and specificity for preoperative detection of at-risk corneas.

#### 3.7.2. Cross-Linking (CXL)

Cross-linking (CXL) is a minimally invasive procedure which presumably induces the formation of new molecular bonds between the corneal collagen fibrils and lamellae using riboflavin and UV light [93]. This procedure of reinforcing the collagen meshwork with CXL has shown to be effective in the treatment of surgically induced ectasia and in halting progression of keratoconus [94–96]. In corneal CXL, the cornea is stiffened and a high increase is observed in Young's modulus by nearly 300% [93]. It would be expected that the biomechanical properties of the cornea will change as a result of the treatment, particularly corneal rigidity parameters. Differences in CH and CRF were observed during the first weeks after CXL treatment that returned to baseline values later. The effect of matrix reorganization or CCT changes immediately after the procedure may explain these differences in CH and CRF [97, 98]; however, sustainable changes in CH and CRF parameters alone that can be correlated with the assumed increase in corneal stiffness induced by CXL [34, 36, 97, 99] were not found and the clinical results did not confirm the *ex vivo* results. From the analysis of the new ORA parameters based on waveform signal analysis, a significant increase (35%) in area under Peak 1 and Peak 2 was observed after six months of treatment, suggesting that this can be the result of a modified corneal surface after CXL, which provides better reflectivity due to an improvement of corneal homogeneity [34, 97]. These recent studies seem to indicate that additional parameters derived from signal analysis provide supplemental information to evaluate the potential positive effect of CXL and to measure the long-term effects of this procedure.

#### 3.7.3. Intrastromal Corneal Ring Segments

Intrastromal corneal ring segments (ICRS) are primarily used for the treatment of primary keratoconus [100] and secondary keratectasia following refractive surgery [101]. The insertion of the ICRS induces a flattening of the central cornea by adding extra material within the corneal paracentral area [102], improving regularity of the corneal shape, and preventing additional degradation of vision [103]. Knowledge of the biomechanical properties of the corneal might help to decide the best treatment approach, predict the success of the treatment, and eventually monitor the postsurgical corneal behaviour. No significant differences were found in CH in the short-term (<3rd month) postoperative period [104–106] which may indicate that the ICRS alter corneal curvature without changing the viscoelastic response of the corneal tissue. A study conducted on 20 patients with keratoconus showed a stable corneal flattening and a decrease of the astigmatism with no statistically significant changes in ORA parameters, 18 months after ICRS implantation [107]. Better visual outcomes could be expected for corneas with lower biomechanical corneal resistance due to easier deformation by the ring implantation. Piñero et al. [108] reported significant changes in CH, 6 months after ICRS implantation, and the authors suggested that these changes may limit the prediction of the ring segment effect in the long term. However, this hypothesis could not be confirmed by a recent study [109], contradicting previous results obtained by the same authors. Although the authors claim in the second publication that prediction of visual acuity (VA) by ORA parameters is feasible in the short term, they could not confirm that in the first study using the same follow-up time of 6 months. Regarding CRF value, significant transient decrease was found during the first 3-month period after the femtosecond laser-assisted ICRS implantation with no significant changes thereafter [105]. New waveform parameters such as the amplitude Peak 2 [104], aplhf, uslope11, w11, path11, time1, and deltatime [110] showed significant differences with respect to the preoperative conditions but those changes were not attributed to a modification of the biomechanical properties induced by the treatment but rather to corneal stabilization. Interestingly, from the waveform analysis provided by Ambrosio et al. [111], it has been recently reported that the corrected and uncorrected distance visual acuity improved more as the pre-ICRS implant biomechanical properties were weaker or less resistant before treatment. This might provide useful information to predict the visual outcomes of ICRS implantation in keratoconus [111].

#### 3.7.4. Keratoplasty

Studies that evaluated corneal biomechanics by ORA showed that corneas after penetrating keratoplasty (PK) or deep anterior lamellar keratoplasty (DALK) present weaker CH and CRF than normal corneas [29, 112–114]. Additionally, Yenerel et al. [112] found that CH and CRF were higher in PK eyes than in forme fruste (FF) or advanced keratoconus (KC) eyes and both CH and CRF parameters approach the range of normal eyes after corneal transplantation. On the other hand, Shin et al. [29] analysed the results of 26 subjects that had undergone PK for different reasons (bullous keratopathy, herpes keratitis, trauma, etc.) in one eye and compared the results with the contralateral nonoperated eye. They reported lower CH and higher CRF post-PK compared with the fellow healthy eye, although these differences were not statistically significant. The effect of different keratoplasty techniques showed that post-PK eyes had lower CH and CRF when compared with post-DALK eyes and post-DALK eyes had CH and CRF values similar to normal eyes. This may be due to the action of Descemet's membrane which is preserved in DALK, which acts as a strong foundation for the rest of the corneal stroma which rests above it. Opposite findings were reported by Jafarinasab et al. [115] that found lower values of CH and CRF in the DALK group compared to PK group, but those differences were not observed 30 months after surgery. Differences between the indications for keratoplasty or graft-related differences [116] may explain the difference in the results of different studies.

### 3.8. Ocular Disease

#### 3.8.1. Glaucoma

Differences in CCT have been considered as a risk factor for glaucoma [117, 118] and given the correlation between low CCT and glaucomatous changes in the optic disc, a biological association shared by the cornea, sclera, and lamina cribrosa is conceivable [119, 120]. A number of recent reports have suggested a relationship between CH, CRF, and glaucoma with evidence that CH is lower in glaucomatous eyes compared with normal eyes and eyes with ocular hypertension [3, 7, 42, 45, 90, 121–124]. Furthermore, normal tension glaucomatous (NTG) eyes show the lowest value among glaucomatous eyes according to some studies [121, 125]. Even after pharmacologic IOP lowering, CH was shown to be lower in glaucomatous eyes than in normal eyes [126]. This suggests that eyes with lower CH and/or thinner than normal CCT might exhibit structural weakness [42] and it is possible that CCT and CH could be considered as risk factors for glaucoma, independent of IOP [121, 122, 127]. Conversely, CRF was found to be significantly higher in patients with ocular hypertension and in patients with primary open-angle glaucoma and low in NTG patients [90, 123]. This implies that GAT IOP should be expected to be overestimated as a greater force required to applanate a cornea with higher CRF. This could suggest that CRF could be also useful to differentiate between subjects with ocular hypertension and glaucoma [123].

As both the sclera and the cornea are formed from continuous extracellular matrix, this might have some effect on the biomechanical relationship between the two tissues [128]. Bochmann et al. [120] compared CH in glaucomatous eyes with and without acquired pit of the optic nerve and reported that CH was lower in glaucomatous eyes with an acquired pit and hypothesized the possibility that corneal biomechanical properties reflect the attributes of the lamina cribrosa [120, 121]. Several studies found that eyes with low CH are associated with increased severity of glaucomatous visual field defects [45, 122, 129, 130]. In contrast, Wells et al. [124] found a relationship between CH and deformation of optic nerve head with higher CH being strongly correlated with higher deformability of the optic nerve head. In untreated newly diagnosed POAG patients, CH was the only factor significantly associated with both mean cup depth (*r* = −0.34) and cup-to-disc ratio (*r* = −0.41) [131].

In conclusion, as the elastic properties of the cornea are believed to reflect the elasticity of collagen fibres in the eyeball as a whole, there might be an opportunity to consider corneal biomechanics as an indicator of overall globe biomechanical properties in glaucoma [132]. If this is true, corneal biomechanical properties seem to be a promising addendum to the complex issues of glaucoma and may constitute a pressure-independent risk factor for glaucoma detection, prognosis, and treatment.

#### 3.8.2. Keratoconus

In keratoconus (KC), the normal corneal collagen-fibril meshwork is disrupted leading to a localized reduction of corneal radius of curvature and tissue thinning. A significant weaker stress versus strain response in KC eyes compared to normal eyes and a more disorganised collagen fibber network as well been shown [16, 133]. Thus, changes in corneal biomechanics in KC eyes might be expected and it has been suggested that KC progression is characterized by a reduction of material properties that lead to a progressive thinning, increasing strain and stress redistribution, and lower keratocyte densities [134, 135]. CH and CRF measurements have been shown to be reduced in KC eyes [4, 28, 39, 85, 136–138] with stronger decrease as KC severity increases [110, 139–141] even after controlling for differences in age, sex, and CCT [141, 142]. This suggests that other structural alterations different from CCT lead to lower lamellar adhesion and lower shear modulus and may be responsible for these lowering effects in ORA measurements [143]. However, there is large overlap of CH and CRF between normal and KC corneas and both ORA parameters showed low sensitivity and specificity in differentiating KC or suspecting KC from healthy corneas [137, 138, 140, 142, 144, 145]. Recent studies demonstrated that the new parameters derived from waveform analysis of ORA signals represent a significant improvement in detection and differentiation of the keratoconic cornea [28, 92, 110, 145, 146]. In fact, characteristics of the air pressure corneal deformation profile are more affected by keratoconus than the traditionally extracted CH and CRF factors; keratoconic eyes have significantly lower elasticity coefficient compared to normal eyes [92] and the area under the second peak of the signal curve has been shown to produce the best results and seems more promising in distinguishing between normal and KC eyes [110, 137].

#### 3.8.3. Fuchs Corneal Dystrophy

Fuchs corneal dystrophy (FCD) is a genetic disorder of the corneal endothelium. When the disease progresses, the number of endothelial cells decreases and corneal oedema increases affecting visual acuity [147]. Both CRF and CH parameters were found to be lower in FCD eyes compared to normal eyes [3, 51, 148]. del Buey et al. [51] reported that CRF was positively correlated with CCT in control eyes while this correlation was negative in FCD eyes. According to the authors, these results may be related not only to corneal hydration but also to other aspects of corneal biomechanics since patients with FCD have decreased endothelial cell density and thicker Descemet's membrane, and the corneal central region is usually involved which can lead to reductions of viscous damping within corneal tissues and, consequently, viscosity reduces. Additionally, the authors found that the lower the CH, the higher the IOPcc in FCD eyes, but these results may be due to an underestimation error in IOP measurement caused by the observed diminished CH and elevated CCT [51]. Similar results were reported by Clemmensen and Hjortdal [148] who found a CH and CRF reduction in FCD eyes and that IOPcc appears to overestimate IOP in those patients. Altogether, corneas affected by FCD point to a paradoxical condition in which thicker corneas are not related as expected to higher CRF as shown in normal eyes. This might also point to a mechanistic explanation to interpret CRF values. According to this, CRF increases with increase in CCT as long as this increase is justified by an increase in collagen material. Conversely, when the increase is due to a massive hydration of cornea as in FCD, the effect is the opposite as the ground substance becomes more relevant in the overall context of the mechanical behaviour of the cornea.

### 3.9. Systemic Disease

#### 3.9.1. Diabetes

Several structural changes in the cornea of diabetes patients have been reported [149, 150] and an influence on the biomechanical properties of the cornea could also be hypothesized. Several studies have investigated the impact of diabetes on corneal biomechanical parameters; however, the results are rather controversial among different studies [151–156]. Goldich et al. [154] found that CH, CRF, and CCT were significantly higher in diabetic eyes compared to healthy eyes. Hager et al. [155] reported a significantly higher CH in diabetic eyes than in nondiabetic eyes after correcting for age, IOP, and CCT. By contrast, Şahin et al. [156] reported that CH was significantly lower in diabetic patients, whereas CRF was not significantly different from that of control subjects. The authors hypothesized that lower CH in diabetic patients may be explained by a decrease in the dampening effects of the cornea as a result of an alteration in the collagenous components in diabetic eyes due to collagen cross-linking. The reasons for such contradictory results among different studies lie in the differences in age range and CCT and diversity of diabetes types and severity enrolled. In some studies most patients presented type 2 diabetes, while in others there were a similar number of patients with type 1 and type 2 of diabetes. In fact, as recently shown by Scheler et al. [152], biomechanical properties of the cornea seem to be altered depending on the glucose control. In their study, Scheler et al. found that in diabetes, CH and CRF were significantly correlated to glycated haemoglobin (HbA1c); diabetic patients with elevated HbA1c showed an increased CH indicating an increase in the viscosity of the ground substance that is associated with higher corneal shearing strength and increased damping most likely due to a nonenzymatic glycosylation of proteoglycans and glycosaminoglycan that affects the corneal damping behaviour [152].

## 4. New Imaging Techniques to Measure the Corneal Biomechanical Properties

Given the promising nature of the possibility of measuring corneal biomechanics *in vivo*, there has been an increasing interest in the development of methods that allow minimally invasive mechanical test of the cornea which may permit a better understanding of the differences in corneal properties between a wide range of ocular conditions and healthy eyes as well as an improvement in the early detection of potential problematic corneas. Until now, many studies covering measurement of corneal biomechanical properties in a wide range of topics have been performed and published using the ORA device as previously described, but other new *in vivo* techniques of corneal biomechanical measurements are under development. However, with the exception of the Corvis ST, most of these new noninvasive or minimally invasive techniques are experimental prototypes that despite being promising still have many drawbacks such as not being commercially available, being of high costs, and lacking evidence of accuracy and availability for clinical purposes that need to be overcome.

One technique is the *Corneal Visualization Scheimpflug Technology (Corvis ST; Oculus, Wetzlar, Germany)* which is commercially available since 2011. This device is based on a noncontact air puff tonometer combined with an ultrahigh speed Scheimpflug camera. The Scheimpflug camera records 4330 images per second along an 8 mm horizontal corneal coverage during corneal deformation under an air puff indentation [157]. This camera allows a dynamic inspection of the deformation process of the cornea and provides further detailed information of biomechanical characterization of the cornea. The Corvis ST output parameters include time and length of the flattened cornea in the first applanation; corneal velocity during the first applanation moment; time from start until the second applanation; length and corneal velocity during the second applanation moment; time from start until the highest concavity of cornea is reached; and maximum deformation amplitude (from start to the highest concavity) at the corneal apex, among others. However, the machine is still under development and new parameters are being continuously added to the output and only available for research purposes. A definition of the parameters currently available in the commercial version of the instrument is provided in Appendix B. Clinical outcomes are limited and preliminary results have found significant differences of corneal deformation response among normal and keratoconic corneas for many parameters such as corneal speed during deformation, corneal applanation length, and deformation amplitude. All of them seem to be relevant parameters to define the corneal stiffness and corneal viscoelastic properties and are promising in the evaluation of several corneal conditions and the outcomes of different surgical procedures [158–161].

Another prototype device is the Dynamic Corneal Surface Topography [162] that involves surface topographic corneal imaging, with a Dynamic Rasterstereographic Corneal Topography (d.RCT) *with off-axis geometry, during an air puff indentation by an NCT* [163]. This device includes an imaging arm, a calibrated grid arm, and a digital camera. When fluorescein is instilled into the cornea and the fluorescent emissions are excited by the projected grid, an image of which is then captured that contains the three-dimensional information from the corneal surface. After approximately 12 ms from the beginning of the air puff, when the air puff pressure is maximum, another image is taken, which corresponds with the largest corneal deformation. From the two images that are acquired (predeformation and middeformation), biomechanical properties can then be determined using a model of corneal viscoelasticity, based on the applied force and the stress-strain relationship of discrete surface segments across the cornea by measuring corneal shape and displacement between the predeformation state and the middeformation state [164].

Another novel method is based on high speed *Swept Source Ocular Coherence Tomography* (ssOCT) combined with an air puff NCT [165]. The cornea is deformed by the air puff, and during the 20 ms of applanation time, the ssOCT acquires multiple A-scans at the center of the air puff, allowing observation of the dynamics of the anterior and posterior corneal surfaces. From the analysis of the scan, one can obtain information about the biomechanical behaviour of the cornea during the applanation process. Pilot results in normal subjects showed the validity of the technique in IOP measurement [165]. However, the system needs improvements particularly in a faster acquisition system and a large clinical study is required to fully understand the potential of the system in the clinical setting.

*Brillouin Optical Microscopy* is another noncontact technique that uses the combination of a confocal microscope with an ultrahigh resolution spectrometer to perform Brillouin imaging of the cornea [166]. It has the ability to visualize corneal elasticity and measure the depth-dependent variation of elastic modulus within the cornea noninvasively with three-dimensional resolution. This device was firstly used in bovine corneas and is currently in development for use in human eyes [166].

Shear wave propagation velocity has been used to measure corneal biomechanical properties *in vivo*, through the use of linear elastic model approximation, in which the Young's modulus and Poisson's ratio can be estimated from the shear wave speed [18, 167, 168]. However, corneal strain and corneal hydration strongly affects the wave speed by attenuating high-frequency shear wave and do not reproduce the nonlinear properties of the cornea. Recently, a new method has been developed: the *Quantitative Ultrasonic Spectroscopy* (QUSi) [169]. The QUSi has improvements in the form of wave propagation that are not available in clinical ultrasound and derives more information of the reflected full-wave forms. Once corneal acoustic and elastic properties have been shown to correlate [170], this method is currently being developed to map corneal elastic properties and so to determine an elastic constant of the cornea called the *aggregate modulus*, which provides a measure of its stiffness [169].

*Corneal Transient Elastography* (CTE) is another technique that is under development for ophthalmologic use and was adapted from a technology in current use for the analysis of breast tissue imaging [171]. It combines the generation of a remote palpation in the cornea and ultrafast (20 000 frames/s) ultrasonic images of the resulting corneal displacements that evolve into a shear wave propagation whose local speed was directly linked to local elasticity. The mainly improvements was at the level of the echographic probe that was specifically designed to couple a homogenous transverse compression wave to the tissue (supersonic mode) and an ultrafast echographic acquisition mode, allowing high resolution and quantitative maps of the whole corneal elasticity [172].

*Optical interferometric techniques* were also used to measure corneal biomechanical properties because they are noncontact, highly sensitive, and capable of simultaneously recording information from across the whole surface. *Holographic interferometry* has been used to assess qualitatively keratoplasty wound integrity *in vivo* [173]. *Electronic speckle pattern interferometry* (ESPI) was used to quantify the effect of microkeratome flap creation on the displacement response of the sheep cornea [174]; however, these techniques are extremely sensitive to environmental disturbances such heat and vibration that may influence its accuracy. *Radial shearing speckle pattern interferometry* (RSSPI) [175] is an interferometric technique where the two images contain information on the topography of the surface location which changes as applied pressure is altered and is much more resistant to physical disturbances. The differential magnification between the two images allows a mathematical analysis to detect changes in radial strain. It has been used to describe the progressive increase in corneal Young's modulus as a function of aging in human corneas [49] and to quantify the magnitude of the stiffening effect of corneal cross-linking [175].

Another technique uses a physical probe to indent the central cornea with an electronically controlled microprecision motor coupled with simultaneous video-topography imaging of the cornea. It is called *Dynamic Corneal Imaging* (DCI) and measures the change in curvature of the cornea as it bends [176]. in this technique, greater difference flexing curves have been demonstrated with lower IOP, thinner corneas, and in keratoconic versus normal corneas as well, which is consistent with more easily deformable corneas [176].

Another technique uses *Optical Coherence Tomography Elastography* [177] to generate *in vivo* 2D maps of corneal deformation as it is indented by a concave curved lens to preserve the curvature of the cornea as it deforms. It has the potential to measure local and depth variations in the mechanical properties of the cornea owing to its ability to measure strain throughout all the stroma, providing measures of local viscoelastic properties such as elastic modulus, shear modulus, and hysteresis [177]. Current efforts include the development of 3D analysis routines and stress sequences for *in vivo* use.

## 5. Conclusions

The published literature sheds light on the potential utility of the biomechanical corneal properties to a better comprehension of the mechanical behaviour of this complex tissue. However, it also shows some to some controversial results in relevant areas such as their impact on intraocular pressure measurement, preoperative refractive surgery assessment, and surgical treatment of keratoconus. New parameters derived from a more detailed analysis of the outcomes as well as new technologies are promising in consolidating the utility of the biomechanical corneal properties as a clinical tool and a very relevant field for the future improvement of safety and efficacy of different eye health care strategies.

## Figures and Tables

**Table 1:** Summary of studies of corneal hysteresis (CH) and corneal resistance factor (CRF) in healthy patients.

Study	Number of eyes	Number of patients	Sample country	Age (years)	CH (mmHg)	CRF (mmHg)
Shah et al. 2006 [50]	207	—	United Kingdom	62.1 ± 18.1	10.7 ± 2.0	10.3 ± 2.0
Kirwan et al. 2006 [38]	91	42	Ireland	(4–18)	12.5 ± 1.4	—
Shah et al. 2007 [39]	207	—	United Kingdom	62.1 ± 18.1	10.7 ± 2.0	—
Lu et al. 2007 [73]	—	20	China	19.7 ± 1.1	11.5 ± 1.4	9.6 ± 1.9
Lam et al. 2007 [24]	—	125	China	23.1 ± 3.3	10.9 ± 1.5	11.0 ± 1.7
Lim et al. 2008 [40]	—	271	Singapore	14.0 ± 0.9 (12–15)	11.8 ± 1.6	11.9 ± 1.7
Touboul et al. 2008 [65]	—	122	France	48.0 (17–80)	10.3	11.1
Song et al. 2008 [59]	—	1233	China	14.7 ± 0.8	10.7 ± 1.5	—
Kirwan and O'Keefe 2008 [6]	84	84	Ireland	36 ± 10	10.8	—
González-Meijome et al. 2008 [68]	58	58	Portugal	22.95 ± 3.92	10.7 ± 1.9	11.4 ± 1.5
Shen et al. 2008 [41]		90	China	33.7 ± 12.4	11.11 ± 1.49	
Chen et al. 2009 [77]	—	20	Hong Kong	24.1 ± 2.6	11.1 ± 1.1	10.7 ± 1.3
Franco and Lira 2009 [30]	63	—	Portugal	33.2 ± 12.2	10.8	10.6
Kamiya et al. 2009 [43]	204	204	Japan	46.7 ± 19.4	10.1 ± 1.5	10.1 ± 1.6
Abitbol et al. 2010 [42]	—	75	France	61.44 ± 10.6 (45–85)	10.46 ± 1.6	—
Kaushik et al. 2012 [90]	—	71	India	>18	9.5 ± 1.4	9.2 ± 1.5

CH: corneal hysteresis; CRF: corneal resistance factor.

**Table 2:** Summary of studies evaluating the influence of refractive surgery on biomechanical parameters.

Author	Sample (eyes)	Outcomes			
		CH (mmHg)		CRF (mmHg)	
Pepose et al. (2007) [25]	66 LASIK	Pre	Post	Pre	Post
		9.7 ± 1.8	8.0 ± 1.6	9.5 ± 1.9	6.7 ± 1.7

Ortiz et al. (2007) [4]	65 LASIK	Pre	Post	Pre	Post
		10.44 ± 1.74	9.3 ± 1.9	10.07 ± 1.97	8.1 ± 1.8

Hamilton et al. (2008) [81]	32 LASIK (flap with femtosecond):	CH decreased 1.9 mmHg			
	33 LASIK (flap with microkeratome):	CH decreased 2.2 mmHg			
	32 PRK:	CH decreased 2.3 mmHg			

Franco and Lira (2009) [30]	63 control	10.8 ± 1.53		10.6 ± 1.71	
	20 LASIK	8.5 ± 1.22		7.7 ± 0.97	

Qazi et al. (2009) [82]		Pre	Post	Pre	Post
	15 LASEK	9.06 ± 1.56	7.16 ± 1.99	8.61 ± 1.76	5.95 ± 2.41
	14 LASIK	10.00 ± 1.77	8.57 ± 2.25	9.87 ± 1.97	7.35 ± 2.49

Kamiya et al. (2009) [84]	36 LASIK	Pre:	10.6	Pre:	10.0
		Post:		Post:	
		1 week:	8.6	1 week:	7.3
		1 month:	9.0	1 month:	7.6
		3 months:	9.0	3 months:	7.8
		6 months:	8.9	6 months:	7.7

Kamiya et al. (2009) [83]		Pre	Post	Pre	Post
	27 LASIK	10.8	8.6	10.3	7.7
	31 PRK	10.8	9.2	10.3	8.4

Shah and Laiquzzaman (2009) [85]	110 LASIK	Pre:	Post:	Pre	Post
		11.4	9.2	10.0	7.6

Chen et al. (2010) [86]	60 LASIK	Pre:	10.59 ± 1.02	Pre:	8.80 ± 1.45
		Post:		Post:	
		1 day:	8.16 ± 0.84	1 day:	5.02 ± 1.16
		10 days:	8.14 ± 0.77	10 days:	4.96 ± 0.98
		1 month:	8.33 ± 0.88	1 month:	5.08 ± 1.31
		3 months:	8.47 ± 0.78	3 months:	5.26 ± 0.96

Ryan et al. (2011) [87]	102 epi-LASIK	Pre:	10.22	Pre:	10.01 ± 1.80
		Post:		Post:	
		1 month:	8.17 ± 1.25	1 month	7.82 ± 1.68
		3 months:	8.46 ± 1.44	3 months:	8.03 ± 1.85
		6 months:	8.63 ± 1.31	3 months:	7.77 ± 1.50
		12 months:	8.53 ± 1.49	3 months:	7.80 ± 1.66

CH: corneal hysteresis; CRF: corneal resistance factor.

## Appendices

### A.

Parameters obtained from signal analysis of the Ocular Response Analyzer:

- P1 area and P2 area: areas under the curves of Peaks 1 and 2, measuring 75% of peaks height.
- P1 area1 and P2 area1: areas under the curves of Peaks 1 and 2, measuring 50% of peaks height.
- *h*1 and *h*2: height of the signal Peaks 1 and 2, measuring 75% of Peaks height.
- *h*11 and *h*21: height of the signal Peaks 1 and 2, measuring 50% of Peaks height.
- *w*1 and *w*2: full width of signal Peaks 1 and 2 at 25% of the maximum of the infrared signal peaks.
- *w*11 and *w*21: width of signal Peaks 1 and 2 at half of the maximum of the infrared signal peaks. These two parameters are also called by other authors FWHM1 and FWHM2 [25].
- aspect1 and aspect2: ratio between width (*w*) and height (*h*) of Peaks 1 and 2, measuring 75% of peaks height.
- aspect11 and aspect21: ratio between width (*w*) and height (*h*) of Peaks 1 and 2, measuring 50% of peaks height.
- uslope1 and uslope2: rate of increase from base (at 25% of maximum of the infrared signal peaks) to Peaks 1 and 2.
- dslope1 and dslope2: rate of decrease from Peaks 1 and 2 (at 25% of maximum of the infrared signal peaks) to base.
- uslope11 and uslope21: rate of increase from base (at 50% of maximum of the infrared signal peaks) to Peaks 1 and 2.
- dslope11 and dslope21: rate of decrease from Peaks 1 and 2 (at 50% of maximum of the infrared signal peaks) to base.
- dive1 and dive2: distance from the first spike of Peaks 1 and 2 to the top of the graph, measuring 75% of peaks height.
- slew1 and slew2: ratio between dive and with (*w*) of Peaks 1 and 2, measuring 75% of peaks height.
- mslew1 and mslew2: the longest continuous line in peaks without a break, measuring 75% of peaks height.
- path1 and path2: absolute value of path length around the peaks, measuring 75% of peaks height.
- path11 and path21: absolute value of path length around the peaks, measuring 50% of peaks height.
- Aindex and bindex: number of times that the peaks change their direction, measuring 75% of peaks height.
- aplhf: high frequency “noise” in regions between peaks (normalized by product of average of peak heights × width of region), measuring 75% of peaks height.

Another parameters that have been also analysed include [28]

- Peak 1 and Peak 2: maximum heights of the corresponding infrared signal peaks,
- PIT: time between Time in and Time out,
- Pmax: maximum value of the air pressure curve,
- TPmax: Time al in which the maximum air pressure occurs,
- DID: damping-induced delay is the time between Time2 and the time corresponding to the symmetrical position of *P*_1_ on Peak 2.

### B.

Parameters obtained from image analysis of the Corneal Visualization Scheimpflug Technology are as follows:

- IOP: an ordinary NCT measurement that is based on the first applanation.
- Pachy: measurement of central corneal thickness (CCT) with optical pachymetry.
- Time of Appl 1 (1st A-time): time from start until the first applanation.
- Length of Appl 1 (1st A length): length of the flattened cornea in the first applanation.
- Velocity of Appl 1 (Vin): corneal velocity during the first applanation moment.
- Time of Appl 2 (2nd A-time): time from start until the second applanation.
- Length of Appl 2 (2nd A length): length of the flattened cornea in the second applanation.
- Velocity of Appl 2 (Vout): corneal velocity during the second applanation moment.
- Time of Hi Con (HC time): time from start until the highest concavity of cornea is reached.
- Deformation amplitude (DA): maximum deformation amplitude (from start to the highest concavity) at the corneal apex.
